# Identification of skin-related lncRNAs as potential biomarkers that are involved in Wnt pathways in keloids

**DOI:** 10.18632/oncotarget.15880

**Published:** 2017-03-03

**Authors:** Xiao-Jie Sun, Qiang Wang, Baofeng Guo, Xian-Ying Liu, Bing Wang

**Affiliations:** ^1^ Department of Plastic and Reconstruction Surgery, China-Japan Union Hospital of Jilin University, Changchun, China; ^2^ Department of Obstetrics and Gynecology, Second Hospital of Jilin University, Changchun, China; ^3^ Department of medication, Second Hospital of Jilin University, Changchun, China

**Keywords:** keloid, long noncoding RNA, Wnt pathway, biostatistics, microarray

## Abstract

The long non-coding RNAs (lncRNAs) regulating encoding transcripts/genes involved in Wnt signalling pathway in keloids is largely unclear. We used a pathway-focused lncRNA microarray to detect the differentiated expression profiles of both lncRNAs and genes involved in Wnt pathway, thus a total of 116 Wnt-targeted genes and 69 Wnt-related lncRNAs aberrantly expressed in keloids were initially identified. A stepwise bioinformatics was further performed to find skin-related lncRNA/gene pairs in Wnt pathway in keloids. Firstly, an lncRNA/gene co-expression network with clustered functional modules was constructed; simultaneously, 114 Wnt-genes regarding to dermis were online enriched using Phenotype Enrichment. Secondly, 17 skin-related keloid-aberrant Wnt-genes were acquired by overlapping the 114 skin-related Wnt-genes with the 116 keloid-aberrant Wnt-genes. Thirdly, after co-expression coefficient of each lncRNA/gene profile being ranked respectively, 11 top co-expressed lncRNAs characterized with the highest co-expression coefficients to the 17 genes were identified. Fourthly, seven of the 11 top co-expressed lncRNAs exhibiting array-detected aberrant expression in keloids, together with their 12 most interactive Wnt-genes, were selected to undergo in-pair intracellularly quantitative PCR validation in keloids. As a result, four lncRNAs including CACNA1G-AS1, HOXA11-AS, LINC00312 and RP11-91I11.1 with their six paired Wnt-genes undergoing both array-and-qPCR as well as lncRNA-and-gene double validation were finally identified as skin-related lncRNA/gene pairs that involved in Wnt signalling pathway in keloids. In conclusion, in-depth exploration on these easily-accessible lncRNAs in keloids might aid to find the novel target on how to maintain highly recurrent tumours benign via Wnt-involved network regulation.

## INTRODUCTION

The Wingless type (Wnt) protein family is composed of 19 secreted glycoproteins [1] which act as key ligands for the Frizzled (FZD) family of receptors, participating in numerous biological functions [2, 3]. Wnt pathway signalling targeted genes (Wnt-genes) have been well-acknowledged to play roles as critical regulators in a wide range of malignant tumorigenesis processes [4] and some skin benign pathological changes [5, 6]. Keloids, also known as keloidal scars, are generally developed in response to wounding and are pathologically a type of fibroblastic tumour characterised by an excess of the extracellular matrix. Keloids are highly recurrent over time after surgical excision, especially without any other combined therapies. Until now, the molecular biogenesis and pathogenesis of keloids has remained largely understudied, and effective therapies for this benign proliferative disease have not been well established [7, 8].

Wnt-genes were also demonstrated to orchestrate proliferation and regeneration in skin wounding response and keloids [9, 10]. However, they remain largely unexplored for the differentiated molecular milieu of Wnt pathways between maintaining excessive proliferation within benign tumours and promoting the limited overgrowth into malignancy. Long noncoding RNAs (lncRNAs) are mRNA-like transcripts of more than 200 nucleotides (nt) in length [11]. Although the molecular regulation of the vast majority of lncRNAs is still unclear, accumulated documents have revealed that lncRNAs play critical roles in various pathogenesis processes, especially in proliferation, differentiation and tumorigenesis [12, 13]. With high crosstalk, lncRNAs have been found to participate in regulating Wnt-related malignant proliferation in colorectal cancer [14], squamous cell carcinoma [15, 16], glioblastoma [17], breast tumours [18], tongue cancer [19] and non-small cell lung cancer [20].

As a highly-recurrent benign dermal tumour, we supposed that in keloids, the lncRNA-dependent regulation of Wnt genes might provide a unique and subtle balance between differentiation and proliferation to keep the excessive cell growth consistently benign. Considering the aforementioned biological features and their relatively easy access for sampling, keloids could be a prospective model for exploring the underlined mechanism on Wnt pathways, which are regulated by lncRNAs and simultaneously regulate numerous target genes in benign and malignant tissues. In the present study, we investigated skin-related lncRNAs in the Wnt pathway in keloids using Wnt-focused lncRNA microarrays, followed by a stepwise biomathematics and intracellular qPCR validation to identify skin-related lncRNAs as biomarkers involved in Wnt-gene regulation in keloids.

## RESULTS

### Expression profile of LncRNAs and gene transcripts that involved in Wnt pathways in keloids

A total of 736 mRNAs/genes/transcripts and 770 lncRNAs, which are involved in Wnt signalling pathways, were detected from the total RNA of four keloid specimens and in-pair normal control skins by using LncPath (Wnt) arrays. Among these detected RNAs, 116 Wnt-genes (70 up-regulated and 46 down-regulated) and 69 lncRNAs (38 up-regulated and 31 down-regulated) were identified as aberrantly and statistically significantly expressed molecules in Wnt signalling pathways in keloids by comparing them to the arrays of normal controls. The complete data set of array-detected profiles are presented in Supplementary Excel 1–4.

### LncRNA/gene co-expression network and functional module detection in Wnt pathways in keloids

**Figure 1:**
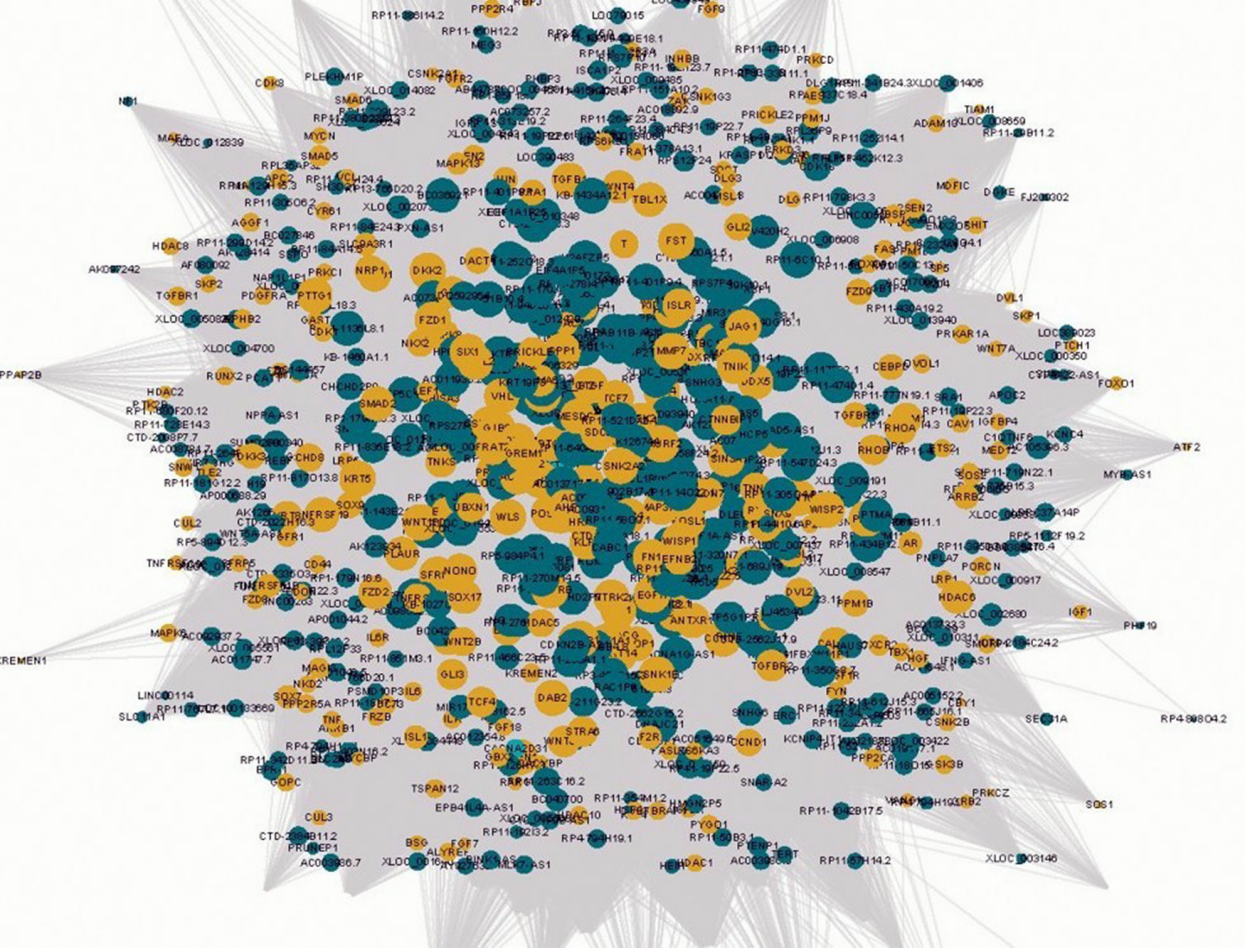
The visualisation of the lncRNA/Wnt gene co-expression network in Wnt pathways in keloids The circular yellow nodes represent the Wnt-genes, and the circular green nodes represent the Wnt-lncRNAs. The node sizes are in proportion to the number of lncRNAs interacting with Wnt genes. The solid lines and dashed lines represent positive and negative correlations, respectively.

To demonstrate the potential interaction between lncRNAs and Wnt-genes in keloids, a co-expression network was constructed based on the pairwise expression correlation between lncRNAs and Wnt-genes (Figure 1, the Original Network can be available as Supplementary PDF1 on request). It consisted of 2.939 nodes (molecules) and 21,751 between-molecule edges in the constructed network with great overlapped interaction. Based on the network, the modules of Wnt-genes in keloids, developed according to clustered functions, were further detected. A topological graph on clustered functional modules with an estimated threshold was produced (Supplementary Figure 1). The Wnt-gene lists in each functional module can also be found in Supplementary Tables 1 and 2.

**Table 1:** The array profile of selected 7 lncRNAs characterized with the top co-expressing coefficients in the network and the significantly differentiation in keloids based on array detection

Molecule	Regulation	Fold Change	*P* value	Chromosome	RNA length	Sequence of single-strand Probe
CACNA1G-AS1	Up	2.5259	0.02756	chr17	1450	CACCAGGGCATATGCAAAGTCACC ATTCTGTGAAATATAAAAGCTC
HOXA11-AS	Up	2.0934	0.00885	chr3	2903	GTGGCACAGCTCATCTTGAAGTAAT TATAATGCCACTAAAATTCTC
LINC00312	Up	1.9706	0.01822	chr9	1628	TCCACAGCCTTTGCAGGCGGAATA TCGGAATAAAGTGGGTCCAGGC
AC004074.3	Down	2.4369	0.00573	chr1	530	GCCCTTCTCCCTCTACAGCTCTGG TGTTACACTGAAAAACTAGAAG
AP001476.4	Down	1.6763	0.00558	chr21	415	GTCACCCACAGCCCTGCCTCTCAG TCACTGCCCGTCACCCACAGCC
RP11-91I11.1	Down	4.0831	0.01469	chr1	151	CCTGTTCCCCAGTGGATTCAGATGA AAACTGGTAATAAAATCAGGT
RP4-794H19.4	Down	1.7152	0.00821	chrX	438	TCAGAGAATTGTAAGTACTCACAGC TTGGCTGAAGATGATGTGGAA

### Identification of skin-related Wnt genes in keloids

**Figure 2:**
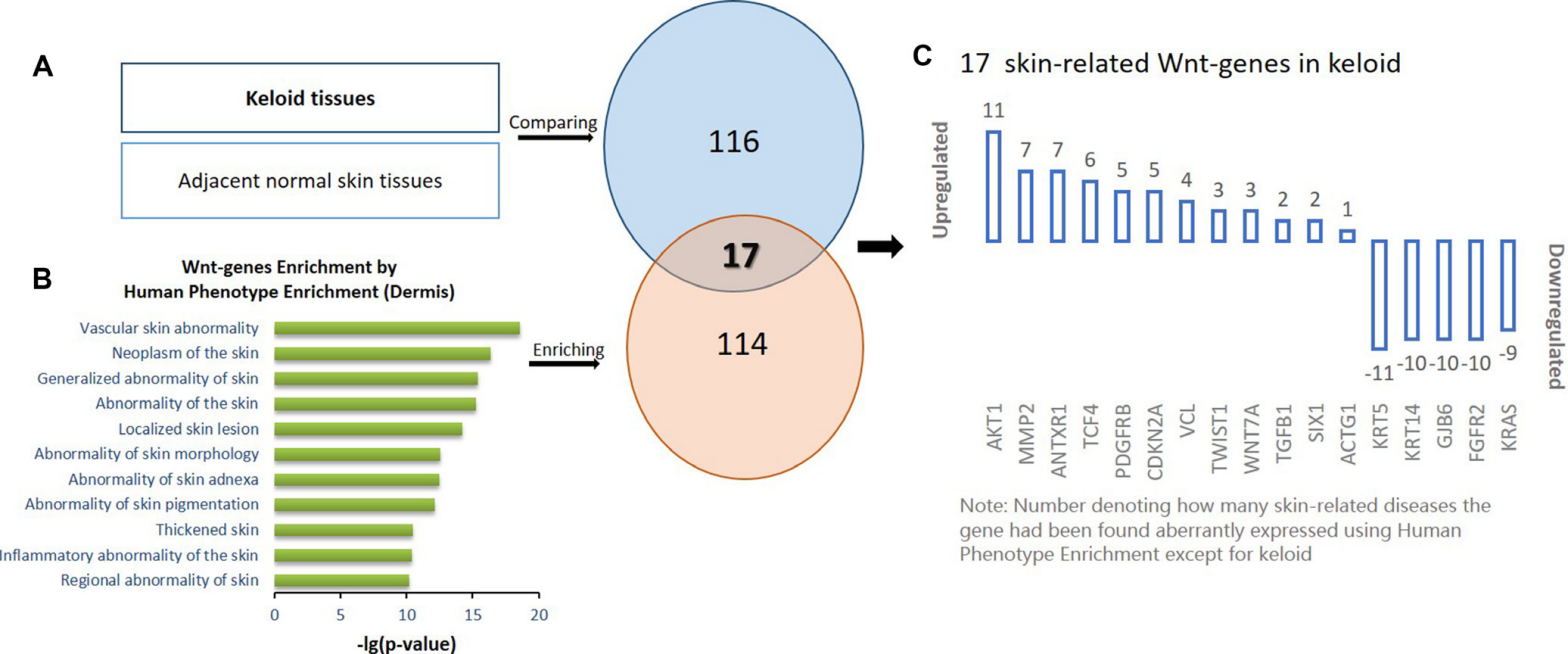
The schematic protocol for stepwise bioinformatics identifying the 17 skin-related keloid-aberrant Wnt-genes (**A**) Identification of the 116 differentially expressed genes involved in Wnt signalling pathway in keloids compared to adjacent normal skin using microarray detection. (**B**) Identification of the 114 dermis-related Wnt-genes by the online Human Phenotype Enrichment from the previous published documents. Based on (A and B), the overlapped 17 skin-related array-aberrant genes involved in Wnt pathway were identified using Venn graphing. (**C**) The number on each of the column indicate how many dermis diseases the gene has been enriched in the previous publication.

Next, the Wnt-genes associated with dermis phenotype were narrowed using the online Human Phenotype Enrichment approach. In total, 114 Wnt-genes which were previously documented to be aberrantly expressed in dermal diseases were identified and described as skin-related Wnt-genes. Using the online Venn graphing program, 17 Wnt-genes overlapped between the 116 keloid-aberrant Wnt-genes and the 114 skin-related Wnt-genes were further identified as skin-related Wnt-genes in keloids (Figure 2).

### Identification of the most interactive Wnt-lncRNAs co-expressing with each of the 17 skin-related Wnt-genes in keloids

**Figure 3:**
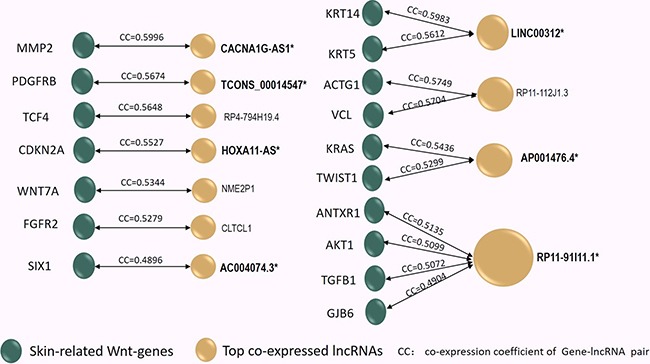
The co-expression coefficient (CC) of the 17 skin-related Wnt-genes (Green Dots) interacting to 11 top co-expressed lncRNAs (Yellow Dots) was in-pair illustrated in the Figure 3 In Supplementary Table 2, the co-expressed lncRNA/gene pair profiles were listed and ranked by CC values, thus the 11 lncRNAs characterized with the top CC to each of the 17 skin-related gene can be identified. Among them, four lncRNAs exhibited overlapped top CC to distinct Wnt-genes (shared Top CC). Notably, among these 11 top interactive lncRNAs, only 7 lncRNAs (Marked by Bold*) displayed significantly alteration due to array detection in keloids. Reversely, the 12 skin-related Wnt-genes (marked by **) were identified as their paired genes. In the next step, the 12 lncRNA/gene pairs (molecular pair marked by genes**/lncRNA*) were selected to undergo intracellular qPCR verification.

Based on the entangled lncRNA/gene co-expression network, each of the 17 skin-related Wnt-genes was found to be co-expressing with a list of at least a dozen lncRNAs (Supplementary Table 3). The most interactive lncRNA, which displayed the highest co-expression coefficient (CC), was selected for each of the 17 skin-related Wnt-genes, thus constructing 17 skin-related gene/lncRNA pairs (17genes/11lncRNAs) in keloids (Figure 3). As seen in Figure 3, we found only 11 lncRNAs were identified to 17 skin-related Wnt-genes due to the overlapping top interaction. Moreover, only the expression of 7 lncRNAs among 11 aforementioned lncRNAs with top CC were found to be significantly changed due to the array detection (marked as Bold* in Figure 3). Therefore, the seven lncRNAs, which were characterised as both top-interactive and array-differentiated, were taken as the candidate lncRNAs in the subsequent intracellular qPCR validation. The array-detected results of the seven selected candidate lncRNAs are shown in Table 1.

### Quantitative validation of the 7 candidate lncRNAs and their paired Wnt-genes by qPCR in keloids

**Figure 4:**
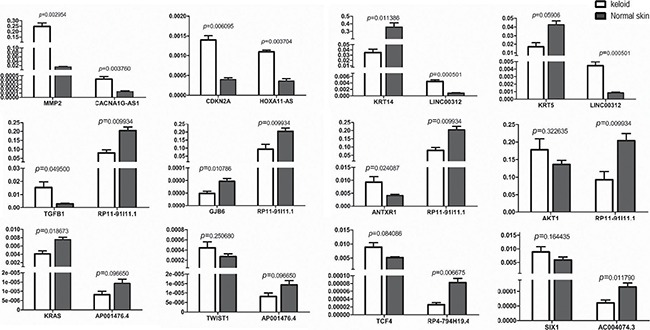
The qPCR results of 7 candidate lncRNAs paired with their top co-expressed Wnt-genes in keloid tissues compared to the adjacent normal skin measured in the validation set

Reviewing to the top co-expressed lncRNA/gene pairs in Figure 3, we found 12 skin-related Wnt-genes (marked as**) identified as the paired genes to the seven candidate lncRNAs. In general, the 12 candidate lncRNA/gene pairs included four one-lncRNA-to-one-gene pairs (CACNA1G-AS1/MMP2, HOXA11-AS/CDKN2A, AC004074.3/SIX1 and TCONS_00014547/PDGFRB), two one-lncRNA-to-two-genes pairs (LINC00312/KRT14, LINC00312/KRT5, AP001476.4/KRAS and AP001476.4/TWIST1) and one one-lncRNA-to-four-genes pair (RP11-91I11.1/ANTXR1, RP11-91I11.1/AKT1, RP11-91I11.1/GJB6, RP11-91I11.1/TGF1, RP4-794H19.4/TCF4). Next, we qualified the expression of these candidate lncRNA/Wnt-gene pairs in keloids by reverse transcriptional qPCR. Primers of 12 lncRNA/gene pairs (7 candidate lncRNAs and 12 candidate Wnt-genes) were listed in Supplementary Table 4. The statistical differences in expression levels of the 12 lncRNA/gene pairs between keloids and the normal skins are presented with *P* value in Figure 4.

Among the 12 lncRNA/Wnt-gene pairs undergoing intracellular qPCR measurement, only 6 lncRNA/gene pairs were identified as successful double validation. It meant, transcriptional levels of three array-up-regulated lncRNAs and three of their paired Wnt-genes, named CACNA1G-AS1/MMP2, HOXA11-AS/CDKN2A and LINC00312/KRT14, were significantly changed in keloids by intracellular qPCR measurement, simultaneously. For the remained array-downregulated candidate lncRNAs, only RP11-91I11.1, with its three paired Wnt-genes, was found significantly altered by intracellular qualification, simultaneously. The remaining six lncRNA/Wnt-gene pairs were not successfully validated for not displaying both array-and-qPCR as well as lncRNA-and-gene significant changed expression in keloids. AKT1, the fourth Wnt-gene paired to RP11-91I11.1, did not significantly change in keloids by intracellular qPCR and therefore did not pass the validation. Neither the expression of KRT5, TWIST1, TCF4 nor that of SIX1 were significantly regulated by intracellular qPCR; therefore, despite the paired lncRNA expression of LINC00312, RP4-794H19.4 and AC004074.3 being successfully validated in keloids by intracellular qPCR, no significant lncRNA/gene pairs could be identified. The expression of lncRNA AP001476.4 was the only lncRNA in keloids that significantly changed by array detection but did not show any statistically significant change by intracellular qPCR, despite the expression of one of its paired Wnt-genes, KRAS, being significantly down-expressed in keloids.

## DISCUSSION

By applying a Wnt-focused array detection followed by step-by-step biomathematics, we identified skin-related Wnt-genes and their most interactive lncRNAs in keloids. The Wnt-focused lncRNA-gene microarray (WntPath™) which is currently in use is aimed to focus on the differentiated expression of lncRNA/genes that are involved in Wnt signalling pathways, by which an increased sensitivity of candidate lncRNA selection was expected. Using a similar strategy, previous researchers have identified 232 differentially expressed lncRNAs related to the Wnt pathway and validated 13 of them by qPCR in lung adenocarcinoma [21]. In the keloids present in this study, we initially revealed that the expression of 69 Wnt-lncRNAs and 116 Wnt-genes was significantly changed by array detection. Furthermore, skin-related lncRNA/gene pairs were validated by intracellular qPCR.

Among the six lncRNA/gene pairs being double-validated, Wnt-lncRNAs CACNA1G-AS1, LINC00312 and HOXA11-AS displayed up-regulated expression in keloids, while the remaining RP11-91I11.1 was the only down-regulated lncRNA in keloids. Of the three up-regulated lncRNAs, the expression of CACNA1G-AS1 was also found to be significantly elevated in keloids using a completely distinct biomathematics analysis based on universal array detection [22]. Therefore, we supposed that CACNA1G-AS1 might be a robust biomarker, based on which, CACNA1G-AS1-dependent Wnt pathway regulation was assumed to play a fundamental role in biogenesis in keloids. To the best of our knowledge, CACNA1G-AS1 has not been previously documented in any other disorders, except in keloids. The second up-regulated lncRNA LINC00312 is also called NAG7. It was initially found to inhibit tumour proliferation by inducing cell apoptosis in nasopharyngeal carcinoma cells [23]. The tumour inhibitory mechanism of LINC00312 was supposed to down-regulate the expression of oestrogen receptor alpha (ER-alpha) by regulating the JNK2/AP-1/MMP1 pathway [24]. Moreover, the expression of LINC00312 was negatively correlated with the size of nasopharyngeal carcinoma, while it was positively correlated with lymph node metastasis [25]. However, in gastric and colorectal cancer, the expression of LINC00312 was not significantly altered [26]. Therefore, we assume that it might be a LINC00312-dependent network which provided subtle yet decisive regulation of cell proliferation and transformation in keloids, which might also be found in other highly recurrent benign tumours. The third most up-expressed lncRNA was HOXA11-AS, which was also documented to increase the progression of glioma [27, 28] and human uterine cervix carcinoma [29, 30]. Also, the functional variant of HOXA11-AS has been revealed to inhibit the oncogenic phenotype of epithelial ovarian cancer [30]. To the best of our knowledge, keloid is the only benign tumour in which HOXA11-AS has been documented aberrantly changed. Therefore, we supposed that HOXA11-AS might also affect the subtle and milieu-dependent regulation of its target Wnt-genes, which help to “confine” the excessive proliferation within the benign phenotype in keloids.

**Figure 5:**
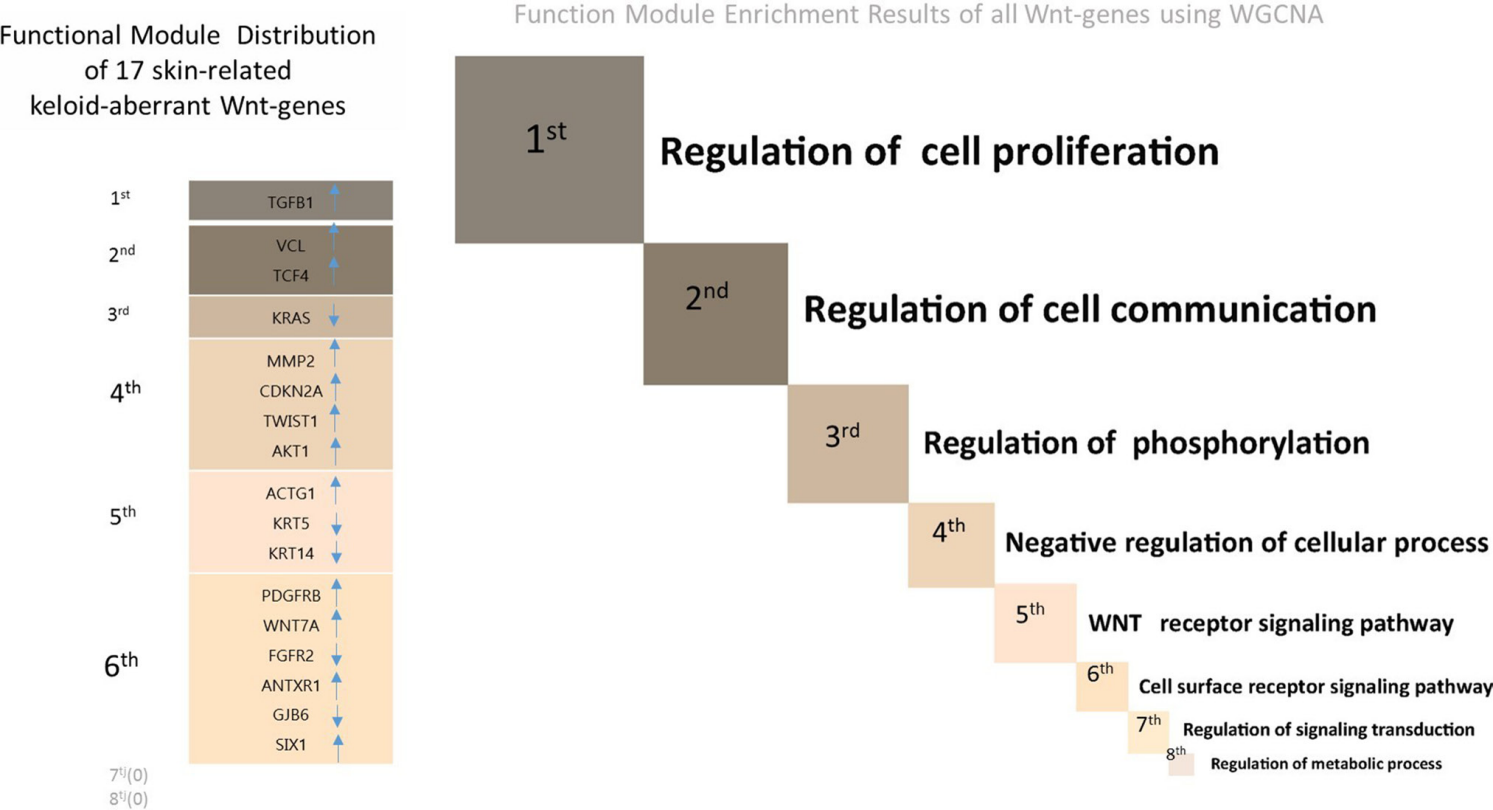
The right part of the figure depicts the eight functional modules (marked from 1^st^ to 8^th^) of all Wnt-genes using the Weighted Correlation Network Analysis (WGCNA) The size of each module is in proportion to the number of clustered Wnt-genes (see Supplementary Table 3 for the detailed gene lists of 8 functional modules). The left part of the figure describes the functional module in which each of the 17 skin-related keloid-aberrant Wnt-genes clustered. The directions of the arrows indicate the array-detected up- or down-regulated expression. Generally, among the 8 functional modules involved in Wnt signalling pathway, 6 skin-related Wnt-genes were clustered in the 6^th^ module (cell surface receptor signalling pathway) which displayed the most clustered module in keloids, followed by 4 skin-related Wnt-genes clustered in the 4^th^ module (Negative regulation of cellular process).

Interestingly, according to our online search results, none of the four down-regulated lncRNAs undergoing PCR validation in the present study have been previously documented in the proliferative disorders. Despite RP11-91I11.1 displaying high overlapping crosstalk with four skin-related Wnt-genes, among which three were successfully validated, no other pathologies relating to RP11-91I11.1 can be searched. However, the highest expression level of RP11-91I11.1, together with its intensive interaction with four skin-related Wnt-genes, indicates that RP11-91I11.1 might also be a potential biomarker warranting in-depth studies in keloids. In addition, we addressed the functional module in which each skin-related keloid-aberrant Wnt-gene was clustered. From the results, it can be seen that most of the 17 selected Wnt-genes were clustered in the lower levels of modules (3^rd^-6^th^) (Figure 5); therefore, we supposed that the lncRNA-dependent gene regulation involved in Wnt pathways might participate in auxiliary regulation within dermis proliferative biogenesis.

In conclusion, we identified four skin-related lncRNAs involved in Wnt-gene regulation in keloids using a step-by-step bioinformatics protocol. We supposed that these four lncRNAs (CACNA1G-AS1, LINC00312, HOXA11-AS and RP11-91I11.1) might play a subtle yet triggering role in the entangled Wnt network in keloids. This skin-focused study could present accessible pathogenesis modelling, which can help to explore how excessive proliferation was exempt from malignancy via Wnt signalling pathways. The in-depth exploration uncovering lncRNA-dependent regulation will be of great value with regard to the Wnt signalling between proliferation and transformation, especially in borderline tumorigenesis.

## MATERIALS AND METHODS

### Preparation of in-pair specimens of keloids

A total of 20 pieces of keloidal scars were obtained anonymously from 20 Chinese patients who underwent cosmetic surgical excision of keloids in the Department of of Plastic and Reconstruction Surgery, China-Japan Union Hospital of Jilin University from January 2016 to March 2016. The paired normal skin samples were simultaneously obtained from the adjacent areas of keloids of the same patient. Among these 20 paired tissues, four pairs were randomly selected for microarray detections and remained 16 pairs were prepared for the further quantitative PCR validation. The usage of surgically removed tissues comply with the ethical rules of Declaration of Helsinki and the present investigation was approved by the Institutional Review Board of Jilin University.

### LncPath (Wnt) RNA microarray assays and differentiated expression analysis

LncPath™ Human Wnt Pathway Microarrays were used to detect array expression of genes that involved in Wnt signalling pathway (Wnt-genes) and lncRNAs that involved in these Wnt-genes (Wnt-lncRNAs) simultaneously in keloids and their adjacent normal skins in-pair from 4 keloid patients. Detailed information of this pathway-focused microarray can be found in http://www.arraystar.com/lncpath-wnt-pathway-microarrays. The hybridization was performed following the protocols of manufacturer. Briefly, total RNA was abstracted, amplified and transcribed into fluorescent cRNA by following the protocol for Arraystar Flash RNA Labeling (Arraystar, Rockville, MD). The labeled cRNAs were hybridized onto the LncPathTM Human Wnt Pathway Array (8×15K, Arraystar). Profiles of Wnt-lncRNAs and Wnt-genes were identified in keloid and normal skin respectively by Agilent Feature Extraction software (version 10.7.3.1). Using a significance analysis of microarrays (SAM) protocol, the differentially expressed Wnt-lncRNAs and Wnt-genes were irrespectively identified based on the cutoff of 1.5-fold change followed by the *P* ranking at greater than or equal to 0.05.

### Stepwise analysis of bioinformatics to screen skin-related Wnt-lncRNAs/Wnt-genes (lncRNA/gene) pairs

To screen skin-related lncRNAs with their potential targeted genes in the Wnt signalling pathway, a step-by-step bioinformatics analysis was performed as described below:

### Construction of the LncRNA/gene co-expression network and detection of the functional modules

The lncRNA/gene co-expression bioinformatics network in the Wnt signalling pathway in keloids was constructed based on the molecular expression correlation using the Weighted Correlation Network Analysis (WGCNA) R software package. To demonstrate the functions of co-expressed Wnt-genes in keloids, an undersigned module detection based on the function of Wnt-regulated genes was clustered by highly interconnected Wnt pathway genes with absolute correlations in the co-expression network. The biological functions of clustered Wnt-genes in the detected functional modules in keloids were annotated by Gene Ontology (GO) functional enrichment (http://david.abcc.ncifcrf.gov).

### Enrichment of previously documented skin-related Wnt-genes

To identify skin-related Wnt-genes in keloids, Wnt-genes within the dermal phenotype were narrowed down using the online Phenotype Enrichment program (http://bejerano.stanford.edu/great/public/html). In total, 435 Wnt-genes which were previously documented as aberrantly expressed in skin disorders were online enriched, and named the skin-related Wnt-gene profile. The skin-related Wnt-genes and keloid-differentiated Wnt-genes by the current array detection were overlapped by an online program (http://www.venndiagram.net) to identify the skin-related Wnt-genes that were also aberrantly expressed in keloids, named skin-related keloid-specific Wnt-genes.

### Identification of the lncRNAs of top co-expressed performance ranked by lncRNA/gene co-expression coefficient to each skin-related keloid-specific Wnt-genes

The profile of lncRNA interacting with each of the skin-related keloid-specific Wnt-genes was individually identified based on the co-expression network. As the lncRNA/gene correlation coefficient, or co-expression coefficient (CC), is supposed to positively relate to the potential interaction intensity that an lncRNA/gene pair performs *in vivo*, in the present study, for each lncRNA profile corresponding to the 17 skin-related Wnt-genes, only the lncRNA of the highest correlation coefficient was selected. Consequently, the most interactive lncRNA/gene pairs with top co-expression coefficient were in-pair identified as the skin-related keloid-specific lncRNA/gene pairs.

### Double validation of skin-related keloid-specific lncRNA/gene pairs by intracellularly quantitative PCR

After the aforementioned skin-related keloid-specific lncRNA/gene pairs involved in the Wnt pathway were identified, the lncRNAs, for which the expression was detected significantly changed in keloids by array analysis, were further selected as validation candidate lncRNAs. Reversely, their paired genes with top co-expressed coefficients were re-identified for the double validation. The candidate lncRNA/gene pairs were in-pair validated by quantitative Polymerase Chain Reaction (qPCR). Candidate lncRNA/gene pairs displaying both array-and-qPCR as well as lncRNA-and-gene significant changed expression in keloids have been considered as successful double validation. The quantitative expression of the candidate lncRNA/gene pairs was individually compared between keloids and adjacent normal skin from 16 patients prepared by qPCR. Total RNA was extracted from the specimens using Trizol reagent (Invitrogen). Reverse-transcription PCR were performed using the PrimeScript RT-PCR reagent Kit (TaKara, Dalian, China) according to the manufacturer's instructions. The expression of β-actin was simultaneously measured as an internal control. The expressed transcripts were measured by qRT-PCR using SYBr Green assays (TaKara). The ratio of expression to β-actin was used to display the intracellular expression of each candidate molecule. Student's *t*-test was used to determine the significance of difference in expression levels between keloids and its adjacent normal skin.

## SUPPLEMENTARY MATERIALS FIGURES AND TABLES











## Abbreviations

Wnt-genes: genes involved in Wnt signaling pathway
Wnt-lncRNAs: lncRNAs related to Wnt-genes
qPCR: quantitative Polymerase Chain Reaction
