# Supplementary material for: Identification of skin-related lncRNAs as potential biomarkers that are involved in Wnt pathways in keloids
Source: Oncotarget. 2017 Mar 3;8(21):34236–44. doi: 10.18632/oncotarget.15880 (PMC5470963; doi:10.18632/oncotarget.15880)
Supplement: Supplementary file 1 [file oncotarget-08-34236-s001.pdf]

Identification of skin-related lncRNAs as potential biomarkers that are involved in Wnt pathways in keloids

Supplementary Materials

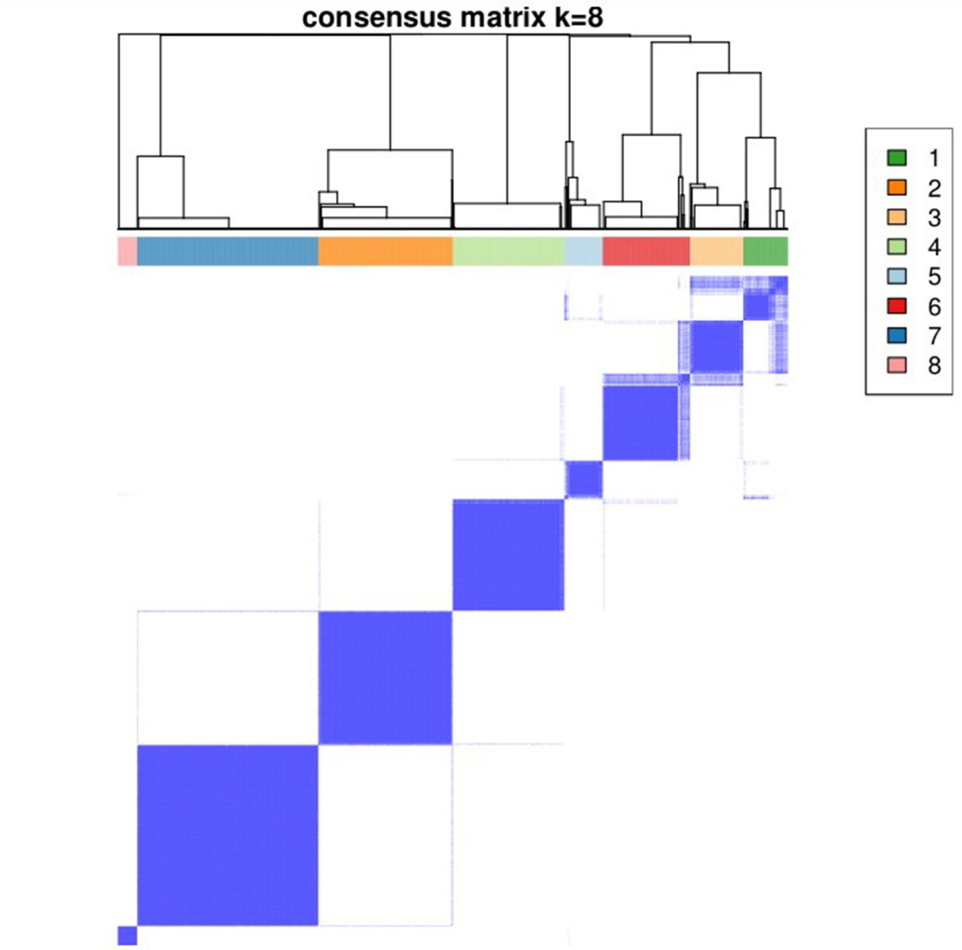

Supplementary Figure 1: Original image produced by R Soft on how genes involving in Wnt pathway clustered in keloid.

Supplementary Table 1: List of clustered functions in each module of the network in Wnt pathway in keloid. See Supplementary_Table_1

Supplementary Table 2: List of Wnt-genes clustered in each functional module of the network in Wnt pathway in keloid. See Supplementary_Table_2

Supplementary Table 3: The interactive profile of lncRNAs to 17 skin-related Wnt-genes ranking by Pearson correlation coefficient. See Supplementary_Table_3

Supplementary Table 4: Primers of validated skin-related lncRNA-gene pairs that involved in Wnt pathway in keloids

Molecules	Sequence of Forward (F) and Reverse (R) Primer	Annealing Temperature (°C)	Primer length (bp)
12 skin-related Wnt genes/mRNAs/transcripts due to reverse network selection			
MMP2	F:5′ GCCCAAGAATAGATGCTGACTG3′<br>R:5′ GAAAGGAGAAGAGCCTGAAGTG3′	60	256
CDKN2A	F:5′ CATTCATGTGGGCATTTCTTG3′<br>R:5′ CTTTGGTTCTGCCATTTGCTA3′	60	140
KRT5	F:GATGAAATCAACAAGCGTACCA<br>R:ACCTCAGCGATGATGCTATCC	60	248
KRT14	F:5′ GAACAAGATTCTCACAGCCACAG3′<br>R:5′ TCTCCACATTGACATCTCCACC3′	60	296
TCF4	F:5′CAGCCATTCTCTTCTGCCAAA3′<br>R :5′ CAGGTTCTCATCACCCTCGTC3′	60	181
ANTXR1	F:5′CTGCCAGGAGGAGACACTTAC3′<br>R :5′ TTTTCATAATAAATCTGCTCACTG3′	60	68
GJB6	F:5′GGGTGACGAGCAAGAGGACTT3′<br>R :5′ TGCGAGTGGTTTCGTGCC3′	60	182
SIX1	F:5′TGCCTAAATGTCTTAATGCTC3′<br>R :5′ CAGGTTGCCAGATTCGTT3′	60	287
AKT1	F:5′CACACCACCTGACCAAGATG3′<br>R :5′ GCACCCGAGAAATAAAAACC3′	60	191
TGFB1	F:5′CCGACTACTACGCCAAGGA3′<br>R :5′ CTGAGGTATCGCCAGGAAT3′	60	247
KRAS	F:5′ATTAGTGTCATCTTGCCTCCCT3′<br>R :5′ CAGCAGCAGTAAATCTTATGGTT3′	60	97
TWIST1	F:5′GACAGTGATTCCCAGACGG3′<br>R :5′ GTCCATAGTGATGCCTTTCCT3′	60	190
7 most co-expressed lncRNAs characterized with array-detected significant alteration in keloids			
CACNA1G-AS1	F:5′ CTGGCAAGGTTGAGTAGGCT3′<br>R:5′ TGCTCCCTTCACACGGTCA3′	60	173
HOXA11-AS	F:5′ TAAACCGCCAACAGCCAGAA3′<br>R:5′ GGGAAGATGAGGGGAGAGG3′	60	107
LINC00312	F:5′ GGTCAGTCCTATGCTTATCCCTT3′<br>R:5′ ATGCTTTGCTCCTGTTGCTCT3′	60	194
RP4-794H19.4	F:5′TCTAAGGACCACTCTATTCACCC3′<br>R :5′GATGTAGCCAGCAGGAAAGCC3′	60	117
AP001476.4	F:5′AGCAGCCGCACTAAGACACT3′<br>R :5′GTGTTTGAACGCGAGAGGGT3′	60	88
RP11-91I11.1	F:5′AAGCAAAATCGTCCTGTTCCC3′<br>R :5′CCCAGCTTAGTTCTTCTCCAATG3′	60	98
AC004074.3	F:5′GCGGGACCCTTTTTATTGC3′<br>R :5′CCTGTTCTTGAAGTTGGGGTC3′	60	74
Internal molecular reference			
β-actin	F:5′ GTGGCCGAGGACTTTGATTG3′<br>R:5′ CCTGTAACAACGCATCTCATATT3′	60	73

Supplementary Excel 1: LncRNA expression profiling data. See Supplementary_Excel_1

Supplementary Excel 2: coding RNA expression profiling data. See Supplementary_Excel_2

Supplementary Excel 3: Differentiated LncRNA expression profiling data. See Supplementary_Excel_3

Supplementary Excel 4: Differentiated coding RNA expression profiling data. See Supplementary_Excel_4
